# Supplementary material for: Patterns of Intron Gain and Loss in Fungi
Source: PLoS Biol. 2004 Nov 30;2(12):e422. doi: 10.1371/journal.pbio.0020422 (PMC532390; doi:10.1371/journal.pbio.0020422)
Supplement: Table S1 — Also available at http://genes.mit.edu/NielsenEtAl/. (4.3 MB ZIP). [file pbio.0020422.st001.zip › NielsenEtAl/html/381.html]

AN4102.1.NCU08755.1.MG09272.1.FG06605.1


```
 CLUSTAL W (1.82) Multiple Sequence Alignments - Introns Inserted


Sequence 1: NCU08755.1	875 aa
Sequence 2: MG09272.1	873 aa
Sequence 3: FG06605.1	886 aa
Sequence 4: AN4102.1	825 aa
Alignment Length: 895 aa
Number Identitical Residues: 370 aa
Alignment Score (without introns) 20024


MG09272.1 	MRFSGIVATLVAGAGVSAHPG----DYS~K0LERRAVATSEPHYPQPWMNPDADGWQEAY
NCU08755.1	MKFAIPLALLASGNLALAAPEPIHPSHQ~Q0LNKRSLAYSEPHYPSPWMDPKAIGWEEAY
FG06605.1 	MKANWLAAAVYLAAGTDAAVPDTLAGVN0L~VARDTLAHSPPHYPSPWMDPNAVGWEDAY
AN4102.1  	MKLGWLEAAALTAASV---------ASA~Q0VKQDDLPVSPPYYPSPWSNGEGE-WAEAY
          	*:     *    .  .               : :  :. * *:**.** : ..  * :**

MG09272.1 	VKAKDFVSQMTLLEKVNLTTGVG2WASDLCVGNVGAVPRLGLRSLCLQDSPTGVRFA~DW
NCU08755.1	EKAKAFVSQLTLLEKVNLTTGIG2WGAEQCVGQTGAIPRLGLKSMCMQDAPLAIRGT~DY
FG06605.1 	AKAKDFVSQMTLLEKVNLTTGVG2WQGERCVGNVGSIPRLGMRGLCLQDGPLGIRFS~DY
AN4102.1  	NRAVQIVSQMTLDEKVNLTTGTG~Y-------------------------------A1DY
          	 :*  :***:** ******** * :                               : *:

MG09272.1 	VSVFPAGITTGATFDKGLMYRRGQAMGQEAKDKGINVLLGPVAGGLGRVAAG1GRAWESF
NCU08755.1	NSVFPAGVTTAATFDRGLMYKRGYALGQEAKGKGVTVLLGPVAGPLGRAPEG~GRNWEGF
FG06605.1 	NSAFPTGVTAGASWSKALWYERGRLMGTEFKEKGIDIALGPATGPLGRHAAG~GRNWEGF
AN4102.1  	NSAFPAGVNVAATWDRQLAYIRGHAMGQEFSDKGIDVQLGPAAGPLGRFPDG~GRNWEGF
          	 *.**:*:...*::.: * * **  :* * . **: : ***.:* *** . * ** **.*

MG09272.1 	GADPVLTGYGMIETIKGIQDTGVIATAKHFIGNEQE1HFRQVGEERGRGVNISESLSSNI
NCU08755.1	STDPVLTGIAMAETIKGTQDAGVVACAKHFIGNEQE~HFRQVGESQDYGYNISETLSSNI
FG06605.1 	TVDPYAAGHAMAETVKGIQDSGVIACAKHYIANEQE1HFRQRGDVMSQKFNISESLSSNL
AN4102.1  	SPDPVLSGVLFAETIKGIQDAGVIATAKHYLLNEQE~HFRQVPEANGYGYNITETLSENV
          	  **  :*  : **:** **:**:* ***:: **** ****  :  .   **:*:**.*:

MG09272.1 	DDKTMHELYLW2PFADAVRAGVGSVMCSYTQVNNSYGCQNSKLLNGLLKDELGFQGFVMS
NCU08755.1	DDKTMHEMYLW~PFVDAIRAGVGSFMCAYTQANNSYSCQNSKLLNNLLKQENGFQGFVMS
FG06605.1 	DDKTMHELYNW~PFADAVRAGVGSIMCSYNQVNNSYACQNSKLLNGILKDEMGFQGFVMS
AN4102.1  	DDKTLHELYLW~PFADAVRAGVGAIMCSYQHLNNTQACQNSHLLNKLLKAELGFQGFVMS
          	****:**:* * **.**:*****:.**:* : **: .****:*** :** * ********

MG09272.1 	DWQAQHTGAASAAAGLDMSM0PGDTEFNTGLSFWGANLTLAVVNGTVAEWRIDDMAMRIM
NCU08755.1	DWQAHHSGVASAAAGLDMSM~PGDTMFNSGRSYWGTNLTLAVLNGTVPQWRIDDMAMRIM
FG06605.1 	DWQAQHTGAASAVAGLDMTM~PGDTEFNTGFSFWGGNLTLAVINGTVPAWRIDDMATRIM
AN4102.1  	DWSATHSGVGSALAGMDMTM~PGDIAFNDGLSYYGPNLTISVLNGTVPQWRVDDMAVRVM
          	**.* *:*..** **:**:* ***  ** * *::* ***::*:****. **:**** *:*

MG09272.1 	AAFFKVGNTLDQPE-INFSSWTKDTFGPLHSSSGN-RIQQINQHVDVRRDHGNLIREVAA
NCU08755.1	AAFFKVGQTVEDQEPINFSFWTLDTYGPLHWAARK-DYQQINWHVNVQGDHGSLIREIAA
FG06605.1 	AAFFKVGRSVEEEPDINFSAWTRDEYGFVQTYAQE-NREKVNFAVNVQHDHKRHIREAGA
AN4102.1  	AAFYKVGRDRLATP-PNFSSWTRAEKGYEHASIDGGAYGTVNEFVDVQQDHASLIRRVGA
          	***:***.        *** **    *  :     .    :*  *:*: **   **. .*

MG09272.1 	KGTVLLKNTNNALPLN-KPKFLAVIGDDAGSNPRGPNGCPDRGCLLGTLGMAWGSGTADF
NCU08755.1	RGTVLLKNTG-SLPLK-KPKFLAVIGEDAGPNPLGPNGCADNRCNNGTLGIGWGSGTGNF
FG06605.1 	KGSVVLKNTG-SLPLK-KPQFLAVIGEDAGSNPAGPNGCADRGCDNGTLAMAWGSGTSQF
AN4102.1  	DSIVLLKNEG-SLPLTGKERNVAILGEDAGSNPYGANGCDDRGCAQGTLAMGWGSGTANF
          	 . *:*** . :***..* : :*::*:***.** *.*** *. *  ***.:.*****.:*

MG09272.1 	PYLITPDAALQAQAIEDGTRYESILSNYATAQTQALVSQTYA~TAIVFVAASSGEGY1ID
NCU08755.1	PYLVTPDQALQARAVQDGSRYESVLRNHAPTEIKALVSQQDA~TAIVFVNANSGEGF~IE
FG06605.1 	PYLVTPDQGISLQAIQDGTRYESILNNNQWPQTQALVSQPNV~TAIVFANADSGEGY~IE
AN4102.1  	PYLVTPEQAIQQEVLKGRGNVFAVTDNWALDKVNKTASES-T2VSLVFVNAGAGEGF~IS
          	***:**: .:. ..::.  .  ::  *    : :  .*:  . .::**. *.:***: *.

MG09272.1 	FDGNKGDRNNLTLWYDGDSLVKNVSSVCNNTIVVIHSTGPTILTEWYDNPNVTAIVWAGV
NCU08755.1	IDGNKGDRLNLTLWNEGDALVKNVSSWCNNTIVVLHTPGPVLLTEWYDNPNITAILWAGM
FG06605.1 	VDGNYGDRKNLTLWKQGDELIKNVSAICPNTIVVLHTVGPVLLTEWHNNPNITAIVWAGV
AN4102.1  	VDGNEGDRKNLTLWKNGENLIKAAASNCNNTIVVIHSVGAVLVDQFYEHPNVTAILWAGL
          	.*** *** ***** :*: *:* .:: * *****:*: *..:: :::::**:***:***:

MG09272.1 	PGQESGRAITDVLYGRVNPAGRSPFTWGKTRESYGTDVMYKPNNGNEAPQQDYTEGVFID
NCU08755.1	PGQESGNSITDVLYGRVNPSGRTPFTWGATRESYGTDVLYEPNNGNEAPQLDYTEGVFID
FG06605.1 	PGQESGNAIADILYGKTSP-GRSPFTWGRTYDSYGTKVLYKANNGEGAPQEDFVEGNFID
AN4102.1  	PGQESGNSLVDVLYGRVNPNGKSPFTWGKTREAYGAPLLTEANNGNGAPQTDHTEGVFID
          	******.::.*:***:..* *::***** * ::**: :: :.***: *** *..** ***

MG09272.1 	YRHFDQQK------------DEPVYEFGHGLSYTTFEYSNIRVDKAPASEYKPTTGQTIP
NCU08755.1	YRHFDKAN------------ASVLYEFGFGLSYTTFEYSNLKIEKHQVGEYTPTTGQTEA
FG06605.1 	YRHFDRQSPSTNGKSATNDSSAPLYEFGFGLSWTTFEYSDLKVESVSNASYSPSVGNTIP
AN4102.1  	YRHFDRTN------------QTPIYEFGHGLSYTTFKYSNLTVQKLNAPAYSPASGQTKA
          	*****: .               :****.***:***:**:: ::.     *.*: *:* .

MG09272.1 	APVFGANVSKDLSQYTFPSDEFPHIYLFIYPYLNTSSSGEEASRDPK--YGGTAEEFLPP
NCU08755.1	APTFG-NFSESVEDYVFPAAEFPYVYQFIYPYLNSTDMS-ASSGDAQ--YGQTAEEFLPP
FG06605.1 	APTYG-NFSKNLDDYTFPSG-VRYLYKFIYPYLNTSSSAEKASGDVKGRFGETGDEFLPP
AN4102.1  	APTFG--TIGEAEDYVFPDS-ITRVREFIYPWINSTDLK-ESSGDPN--YGWDDEDYIPE
          	**.:*     . .:*.**   .  :  ****::*::.    :* * :  :*   ::::* 

MG09272.1 	KALDGSPQPLPRASGKNSPGGNRQLYDTLYTVTATITNTGKLVGEEVPQL~YVSHGGPED
NCU08755.1	KANDGSAQPLLRSSGLHHPGGNPALYDIMYTVTADITNTGKVAGDEVPQL~YVSLGGPED
FG06605.1 	NALNGSSQPRLPSSG--APGGNPQLWDIMYTVTATITNTGDATSDEVPQL~YVSLGGEGE
AN4102.1  	GAKDGSPQDVLPSGG--GAGGNPRLYDDLFRITAIIKNTGPVAGTEVPQL0YVSLGGPNE
          	 * :**.*    :.*   .***  *:* :: :** *.***  .. ***** *** **  :

MG09272.1 	PPVVLRGFERIR-LDPGQSATFKVDLTRRDVSNWDVKVQDWVISEHPKKVFVGSSSRKLH
NCU08755.1	PKVVLRGFDRLR-VEPGEKVQFKAVLTRRDVSSWDTVKQDWVITEYAKKVYVGPSSRKLD
FG06605.1 	PVRVLRGFERLENIAPGESATFTAQLTRRDLSNWDVNVQNWVITDHAKKIWVGSSSRNLP
AN4102.1  	PKVVLRGFDKLV-IQPGEERVFTTTLTRRDLSNWDMEKDDWVITSYPKKGVRG---KLLT
          	*  *****:::  : **:.  *.. *****:*.**   ::***:.:.**   *   : * 

MG09272.1 	LSADLN
NCU08755.1	LEEVLP
FG06605.1 	LSADL-
AN4102.1  	QASS--
          	
```
